# Germline and somatic *SDHx* alterations in apparently sporadic differentiated thyroid cancer

**DOI:** 10.1530/ERC-14-0537

**Published:** 2015-04

**Authors:** Ying Ni, Spencer Seballos, Shireen Ganapathi, Danielle Gurin, Benjamin Fletcher, Joanne Ngeow, Rebecca Nagy, Richard T Kloos, Matthew D Ringel, Thomas LaFramboise, Charis Eng

**Affiliations:** 1 Genomic Medicine Institute, Lerner Research Institute, Cleveland Clinic, 9500 Euclid Avenue, NE-50, Clevland, Ohio, 44195, USA; 2 Taussig Cancer Institute, Cleveland Clinic, 9500 Euclid Avenue, NE-50, Cleveland, Ohio, 44195, USA; 3 Stanley Shalom Zielony Nursing Institute, Cleveland Clinic, 9500 Euclid Avenue, NE-50, Cleveland, Ohio, 44195, USA; 4 Division of Medical Oncology, National Cancer Center, Singapore, 169610, Singapore; 5 Division of Human Genetics, Department of Medicine, The Ohio State University, Columbus, Ohio, 43210, USA; 6 Division of Endocrinology and Metabolism, Department of Medicine, The Ohio State University, Columbus, Ohio, 43210, USA; 7 Comprehensive Cancer Center, Arthur G. James Cancer Hospital and Richard G. Solove Research Institute,The Ohio State University, Columbus, Ohio, 43210, USA; 8 Department of Genetics and Genome Sciences, Case Western Reserve University School of Medicine, Cleveland, Ohio, 44106, USA; 9 Department of Epidemiology and Biostatistics, Case Western Reserve University School of Medicine, Cleveland, Ohio, 44106, USA; 10 CASE Comprehensive Cancer Center, Case Western Reserve University, Cleveland, Ohio, 44116, USA

**Keywords:** *SDHx*, variant, gene expression, papillary thyroid cancer

## Abstract

Along with breast and endometrial cancers, thyroid cancer is a major component cancer in Cowden syndrome (CS). Germline variants in *SDHB/C/D (SDHx)* genes account for subsets of CS/CS-like cases, conferring a higher risk of breast and thyroid cancers over those with only germline *PTEN* mutations. To investigate whether *SDHx* alterations at both germline and somatic levels occur in apparently sporadic breast cancer and differentiated thyroid cancer (DTC), we analyzed *SDHx* genes in the following four groups: i) 48 individuals with sporadic invasive breast adenocarcinoma for germline mutation; ii) 48 (expanded to 241) DTC for germline mutation; iii) 37 pairs DTC tumor-normal tissues for germline and somatic mutation and mRNA expression levels; and iv) data from 476 patients in the Cancer Genome Atlas thyroid carcinoma dataset for validation. No germline *SDHx* variant was found in a pilot series of 48 breast cancer cases. As germline *SDHx* variants were found in our pilot of 48 thyroid cancer cases, we expanded to three series of DTC comprising a total 754 cases, and found 48 (6%) with germline *SDHx* variants (*P*<0.001 compared with 0/350 controls). In 513 tumors, we found 27 (5%) with large somatic duplications within chromosome 1 encompassing *SDHC*. Both papillary and follicular thyroid tumors showed consistent loss of *SDHC/D* gene expression (*P*<0.001), which is associated with earlier disease onset and higher pathological-TNM stage. Therefore, we conclude that both germline and somatic *SDHx* mutations/variants occur in sporadic DTC but are very rare in sporadic breast cancer, and overall loss of *SDHx* gene expression is a signature of DTC.

## Introduction

Succinate dehydrogenase (SDH) or complex II of the mitochondrial respiratory chain catalyzes the oxidation of succinate to fumarate in the Krebs cycle with electron transfer to the terminal acceptor ubiquinone. The SDH complex has four subunits encoded by autosomal genes *SDHA*, *SDHB*, *SDHC*, and *SDHD* (reviewed in Eng *et al*. (2003)). Germline homozygous or compound heterozygous mutations in *SDHA* result in severe neurological dysfunction, such as Leigh syndrome, a rare but fatal neurodegenerative disease (Bourgeron *et al*. 1995, Parfait *et al*. 2000). In contrast, germline heterozygous mutations in the genes encoding the SDH subunits result in hereditary pheochromocytoma–paraganglioma syndrome (Baysal *et al*. 2000, Neumann *et al*. 2002, 2004). It was noticed that a rare subset of individuals with germline *SDHB* or *SDHD* mutations in the European–American pheochromocytoma registry had renal cancers and papillary thyroid cancer (PTC) (Neumann *et al*. 2004, Vanharanta *et al*. 2004).

Cowden syndrome (CS (MIM 158350)) is an autosomal dominant heritable neoplasia syndrome. Because epithelial thyroid, breast, and renal carcinomas are component to CS (Pilarski & Eng 2004, Tan *et al*. 2012), we wanted to determine whether germline *SDHB/C/D* (*SDHx*) variants could also occur in *PTEN* (MIM 601728) mutation-negative CS/CS-like individuals. Indeed, we found 8–10% of such patients carry *SDHx* mutations (Ni *et al*. 2008, 2012). CS/CS-like individuals with germline *SDHx* variants have significantly higher risks of developing breast cancer and epithelial thyroid cancer compared with *PTEN* mutation-only carriers. Among all *SDHx* variant carriers with thyroid cancer in CS/CS-like cases, papillary histology is the major subtype in contrast to the over-presentation of follicular histology in *PTEN* mutation carriers (Ngeow *et al*. 2011, Ni *et al*. 2012).

Family history is an important risk factor for epithelial thyroid cancer: case–control studies have consistently shown that the risk to first-degree relatives of probands is three- to 11-fold, being among the highest of all cancers (Risch 2001, Hemminki *et al*. 2005). However, no predisposing genes have yet been detected that account for anything but a small portion of all familial and particularly sporadic cases. In 2014, 62 980 new cases of thyroid cancer will be diagnosed and more than 1890 patients will die from it, a number that is rising yearly despite aggressive multi-modal therapy (NationalCancerInstitute). It is worth noting that, in addition to the rising incident in thyroid cancer among all cancers, almost all of the increase is in the papillary histology subtype, the subtype we observed associated with *SDHx* variations in CS.

Taking all these observations together, we sought to address the hypothesis that alterations in *SDHx* at both germline and somatic levels may also occur in apparently sporadic breast cancer and differentiated thyroid cancer (DTC).

## Research participants, materials and methods

### Research participants

Consenting adult patients with invasive female breast adenocarcinomas seen anywhere in the Cleveland Clinic Health System were prospectively accrued. The most proximal 48 research participants diagnosed with breast cancers within this prospective series were enrolled for purposes of our current pilot study of apparently sporadic breast cancers. In parallel, a pilot of 48 then expanded to 241 consecutive patients with differentiated epithelial thyroid carcinoma visiting a multi-disciplinary thyroid tumor clinic at The Ohio State University between August 2006 and September 2007 were prospectively accrued. Individuals were eligible for an IRB-approved research study on individuals with thyroid cancer and completed detailed medical history and family history questionnaires. Participants were also asked to donate a blood sample for genetics research.

Thirty-seven thyroid tumor tissues together with paired adjacent normal tissue samples from Corporative Human Tissue Network (CHTN) are accrued via archival anonymized registry protocol.

Three hundred and fifty normal (population) controls are whites of northern and western European origin and were anonymized before storage and analysis. Informed consent was obtained for all subjects in accordance with procedures and protocols approved by the respective Human Subjects Protection Committee of each participating institution.

### Genomic DNA extraction

Genomic DNA was extracted from snap-frozen tumor and adjacent normal tissues using GeneJET Genomic DNA Purification kit (Thermo Scientific, Waltham, MA, USA).

### *SDHx* mutation and copy number variation analysis

Genomic DNA was first analyzed using high-resolution melting LightScanner technology (Idaho Technology, Inc., Salt Lake City, UT, USA), which detects nucleic acid sequence variations by changes in the melting curve. Primers to amplify a total of 20 amplicons spanning the exons, exon–intron junctions and flanking intronic regions as well as promoters of *SDHB/C/D* were designed using LightScanner Primer Design Software (all primers are listed in Supplementary Table 1, see section on supplementary data given at the end of this article) and optimized according to the manufacturer's instructions. Germline genomic DNA samples were amplified with LCGreen Plus (Idaho Technology, Inc.) in a final reaction volume of 10 μl with 20 μl oil overlay. The temperature cycling protocol consisted of an initial denaturation step at 95 °C for 2 min, followed by 37 cycles of denaturation at 94 °C for 30 s, optimal annealing temperature for each amplicon for 30 s, and heteroduplex formation step at 95 °C for 30 s and final hold at 25 °C. Melting curve analysis was performed on LightScanner with LightScanner software employing three steps, namely, normalization, temperature shift, and generating difference plot to cluster samples. The samples with melting curves that clustered differently from reference samples were directly sequenced for *SDHB*, *SDHC*, or *SDHD*, as previously reported by our laboratory (Mutter *et al*. 2000, McWhinney *et al*. 2004).

Deletion analysis using the multiplex ligation-dependent probe amplification (MLPA) assay (Schouten *et al*. 2002) was performed using the P158 MLPA kit (MRC-Holland, Amsterdam, The Netherlands) according to manufacturer's protocol. Genome-wide SNP array analysis was performed using Illumina HumanOmni2.5 BeadChip containing ∼2.5 million markers. Copy number variation (CNV) was called out by CNV partition plug-in in the Beadstudio Software.

### RNA extraction and quantitative RT-PCR

Total RNA was extracted from peripheral blood-derived lymphoblastoid cell lines from controls and patients using the GeneJET RNA Purification kit (Thermo Scientific), according to the manufacturer's protocol, and subsequently treated with DNase I (Invitrogen). DNase-treated total RNA was reverse-transcribed into cDNA using qScript cDNA SuperMix (Quanta BioSciences, Inc., Gaithersburg, MD, USA) as specified by the manufacturer. Quantitative PCR was performed on LightCycler 480 system (Roche Diagnostics Corporation) using the TaqMan primer-probe ready mix for *SDHB, SDHC, SDHD*, and *18S* as endogenous loading control (Life Technologies).

### The Cancer Genome Atlas dataset analysis

Whole genome and/or exome sequencing (WGS/WES) .bam files were obtained through the Cancer Genome Atlas (TCGA) project via download from the Cancer Genomics Hub (https://cghub.ucsc.edu). Germline data were generated from peripheral blood samples. Patient clinical information and SNP array data were downloaded from TCGA's Data Portal (https://tcga-data.nci.nih.gov/tcga/tcgaDownload.jsp).

From the .bam sequence files, *SDHx* gene reads were extracted using SAMtools (Li *et al*. 2009). The bam2fastq software (http://gsl.hudsonalpha.org/information/software/bam2fastq) was used to revert these aligned sequences to fastq format, which were realigned against hg19 reference genome using bowtie2 (Langmead & Salzberg 2012) and the pileup file was generated using SAMtools. From the pileup file, variants were called using bcftools and the Integrative Genome Viewer (IGV) (Thorvaldsdottir *et al*. 2013).

The CNV data were collected from TCGA genome_wide_SNP_6 level 3 dataset, using ‘nocnv_hg19.seg’ results downloaded from TCGA's Data Portal, which removed CNV in a panel of more than 3000 blood normals from TCGA and used hg19 as the reference genome.

The gene expression analysis from the TCGA dataset was based on IlluminaHiSeq_RNASeqV2 ‘rsem.gene.normalized_results’ files downloaded from TCGA's Data Portal, in which RNA-seq reads were quantified by upper quartile normalized RNA-seq by expectation maximization (RSEM) count estimates. In order to quantify multiple gene expression as a set, we generated a variable gene_score (range from 0 to 4) by first categorizing each gene's expression as either 1 as higher than normal, or 0 as lower than normal, then adding up the binary score for each of these *SDHA–D* genes.

The DNA methylation data from Illumina Human Methylation 450 BeadChip array were downloaded from TCGA's Data Portal, where absolute methylation values (*β*-value) of all available CpG sites for each of *SDHx* genes were extracted for 500 thyroid tumors and 56 normal tissues. Then the average of *β*-values from all CpG sites within a CpG island for each gene promoter region was calculated and used for comparison. The CpG island was annotated using City of Hope CpG Island Analysis Pipeline (COHCAP) (Warden *et al*. 2013) based on Illumina Human Methylation 450k platform with respect to hg19.

### Statistical analysis

Statistical analysis was carried out using SPSS (IBM SPSS Statistics for Macintosh, Version 21: IBM Corp., Armonk, NY, USA) with significance at *P*<0.05.

## Results

### Germline *SDHx* variants in apparently sporadic PTC patients but absent in apparently sporadic breast cancer patients

Breast cancer and thyroid cancer are major malignancies associated with CS/CSL; *SDHx* variant carriers show significantly increased prevalence of both breast and thyroid cancers compared with *PTEN* mutation carriers (Ni *et al*. 2012). Therefore, we sought to determine whether *SDHx* alleles also associate with apparently sporadic breast cancer cases and in apparently sporadic thyroid cancer cases. To pilot this hypothesis, we performed germline mutation scanning in 48 women (median age 45, 37–85 years) with invasive breast cancers. This sample size gave us >80% power to detect a 5% prevalence of *SDHx* variants. No mutation or variant was identified in these 48 breast cancer cases, and so we did not proceed to a validation series or further studies in breast cancer.

When our pilot of 48 apparently sporadic DTC samples revealed germline variation in *SDHB* and *SDHD*, we expanded our series to a total of 241 unrelated *PTEN* mutation negative research participants with differentiated thyroid carcinoma (Nagy *et al*. 2011) from The Ohio State University's (OSU) Thyroid Center. Of the 241, we found 15 (6%) with *SDHB/D* missense variants (*P*<0.001 compared with 0/350 controls), six in *SDHB* (Ala3Gly (*n*=1), and Ser163Pro (*n*=5)), and nine in *SDHD* (Gly12Ser (*n*=6) and His50Arg (*n*=3)) (Table 1). Consistent with what we observed in our CS/CSL series, the *SDHD* variants comprise the major proportion (9/15, 60%) of all variants.

In order to further confirm our findings, we used the TCGA thyroid cancer (THCA) dataset composed mainly of PTC samples as a validation series for germline *SDHx* variation. Of all 476 TCGA PTC patients who had WGS/WES .bam files from peripheral blood-derived DNA, a total of 28 (6%) had germline *SDHx* variants, with 13 in *SDHB* (Ala3Gly: *n*=1, Gly53Glu: *n*=1, Thr60Ala *n*=1, Asp142Val *n*=1, and Ser163Pro: *n*=9) and 15 in *SDHD* (Gly12Ser: *n*=10 and His50Arg: *n*=5) (Table 1). These variants were detected in sequencing data from both blood and corresponding primary tumor samples confirming they are indeed germline. The histological type of these 28 papillary carcinomas includes four of follicular variant form, two of tall cell variant form, and 22 of classical form.

### Somatic *SDHx *alterations in sporadic thyroid tumors

We performed mutation analysis of the *SDHx* genes in 37 pairs of apparently sporadic epithelial thyroid carcinomas with adjacent normal tissue from CHTN. Among these tumors, seven are follicular in histology (FTC), four follicular variant of papillary histology (FvPTC), and 26 classic papillary histology (cPTC). Missense *SDHx* variants were identified in five of 37 pairs of samples (*SDHB* Ala3Gly *n*=2, Ser163Pro *n*=1, and *SDHD* His50Arg *n*=2) (Table 1), in both tumor and paired adjacent normal samples, confirming their germline origin. The five individuals with germline *SDHx* variants had three cPTC and two FTC. Thus, there was an overall 6% prevalence of germline *SDHB/D* variants in the combined datasets comprising 754 thyroid cancer patients compared with 0/350 of our residential population controls (*P*<0.001) (Table 1).

No somatic intragenic *SDHx* variants were detected in our 37 thyroid carcinoma samples. Similarly, no somatic intragenic *SDHx* variants were detected in 476 PTC samples that also had matched blood samples (germline) in the TCGA dataset.

**Figure 1:**
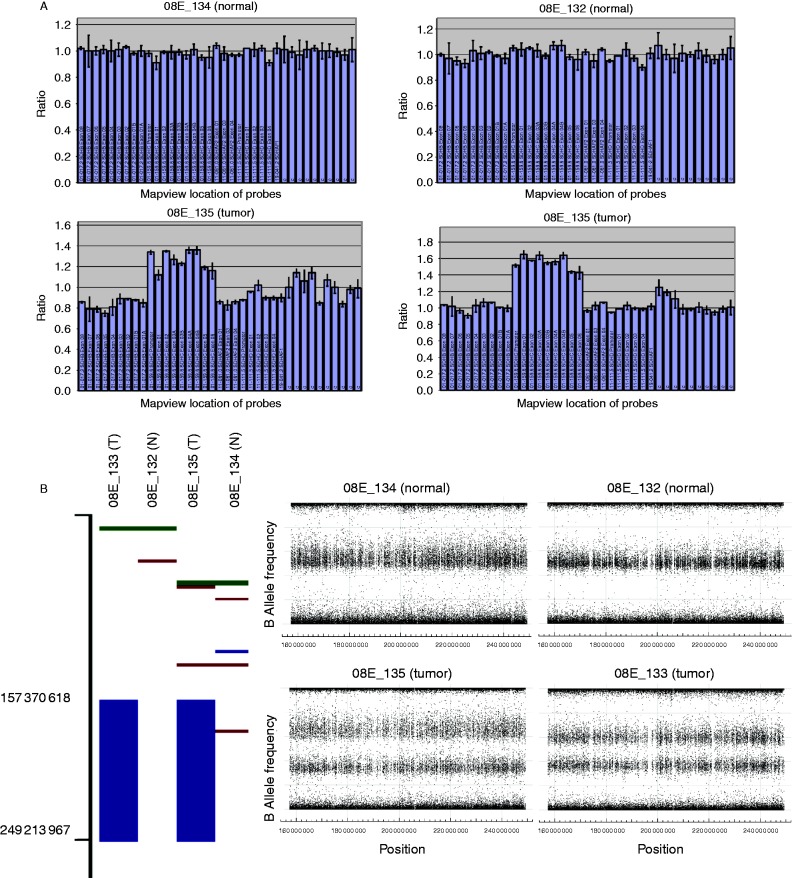
Somatic duplication identified in CHTN papillary thyroid carcinoma samples. (A) Representative MLPA result for two-paired tumor-normal samples: 08E_134-08E_135 as pair and 08E_132-08E_133 as pair; each column represents the relative quantification ratio for each probe included in the kit. (B) Genome-wide SNP array-based CNV analysis on chromosome 1. Left panel showed the large duplication (blue bars) by CNV partition plug-in from GenomeStudio in samples displayed in (A); right panel showed B Allele Frequency plot for the same samples for genomic region 157 000 000–250 000 000.

We then searched for somatic large insertions/deletions of the *SDHx* genes using MLPA in our 37 paired samples. Interestingly, two PTC samples (5%) showed somatic duplication of *SDHC* (Fig. 1A). To further confirm and investigate the size of the CNV region, we subjected both samples with *SDHC* somatic duplication to a genome-wide SNP-array analysis. CNV analysis based on SNP array revealed a three-copy duplication region spanning position 157 370 000–249 213 900 bases on chromosome 1, where *SDHC* is located (chr1: 161 314 376–161 364 751) (Fig. 1B).

CNV analysis of the TCGA THCA dataset revealed 25 of 476 (5%) PTC samples also having somatic duplication (three copies) of the same *SDHC* gene region as we observed in our in-house CHTN tumor samples (Supplementary Table 2, see section on supplementary data given at the end of this article). It is worth noting that the somatic *SDHC* duplication, resulting in three copies of *SDHC* in the genome, did not occur in patients with germline *SDHB/D* variation.

### Overall reduced *SDHx* gene expression in differentiated thyroid carcinoma samples

**Figure 2:**
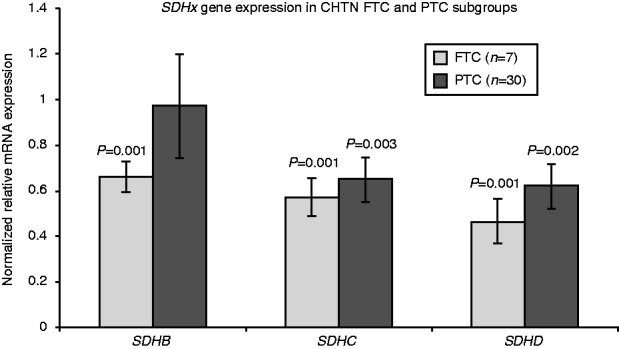
*SDHx* gene expression in FTC and PTC subgroups of CHTN sporadic thyroid samples. Expression was normalized for tumor sample to its paired normal tissue. Data were presented as mean±s.e.m. and *P* value was obtained using two-tailed Student's paired sample *t*-test.

As a pilot, we initially checked the *SDHB/C/D* mRNA expression in our 37 paired sporadic thyroid tumor-normal tissue samples (seven FTC and 30 PTC). Despite the two PTC tumors with *SDHC* duplication showing increased *SDHC* mRNA expression, significantly reduced transcript expression of *SDHC* and *SDHD* was observed in the tumor samples compared with their paired normal tissue (both *P*<0.001). Of note, FTC samples also had decreased *SDHB* transcript expression, whereas there was no change in *SDHB* expression in the PTC samples (Fig. 2).

**Figure 3:**
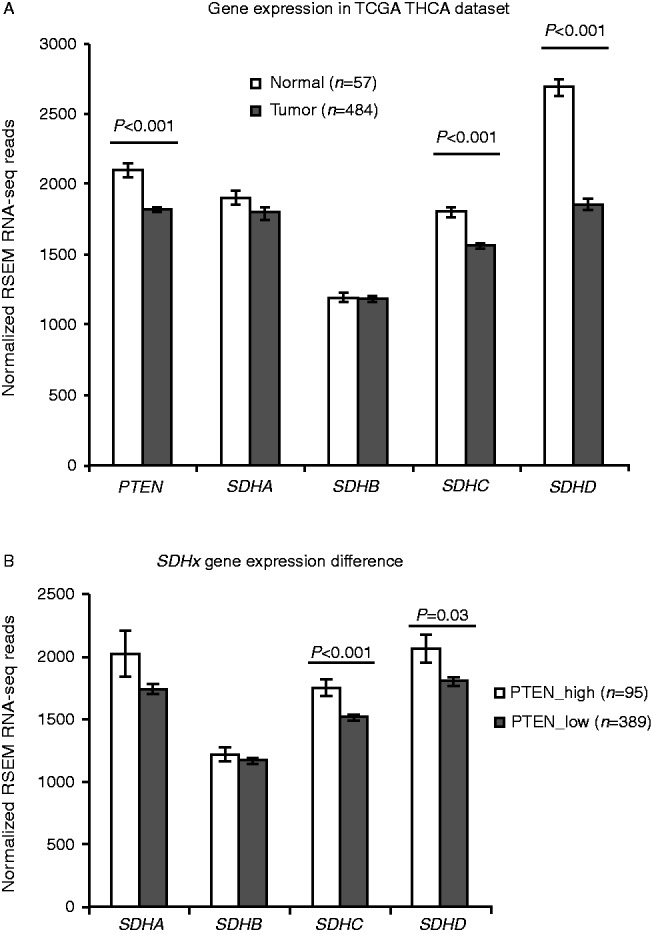
*PTEN* and *SDHx* gene expression in TCGA THCA dataset. (A) *PTEN* and *SDHx* gene expression in tumor samples compared with normal samples; (B) *SDHx* gene expression differences in high *PTEN* expression tumor group compared with low *PTEN* expression tumor group. *P* value was obtained using two-tailed Student's *t*-test.

To survey *SDHx* gene expression in TCGA THCA tumor samples, we extracted normalized RNA-seq read counts for *SDHA–D* and *PTEN* genes. Compared with available normal thyroid tissues (*n*=57), thyroid tumor tissue (*n*=484) showed significant reduction in *PTEN*, *SDHC*, and *SDHD* gene expression (*P*<0.001, Fig. 3A). Interestingly, the transcript expression of each of the *SDHA/B/C/D* genes has strong positive correlations with one another (Supplementary Table 3, see section on supplementary data given at the end of this article). Notably, when we divided the tumors into two groups based on *PTEN* gene expression status (*PTEN*_high and *PTEN*_low, compared with the average of its expression in normal tissue), to mimic the loss of *PTEN* as has been reported in thyroid tumors (Bruni *et al*. 2000, Gimm *et al*. 2000), we found significant reduction in *SDHC* and *SDHD* transcript expression in the *PTEN*_low group compared with the *PTEN*_high group (*P*<0.001 and *P*=0.02 respectively, Fig. 3B). In order to see if the decreased *SDHC/D* gene expression in tumors is caused by DNA hypermethylation, we checked the DNA methylation levels in *SDHC* and *SDHD* gene promoter regions in the TCGA dataset. There was no significant difference in these two genes' promoter methylation levels in tumors compared with normal tissues. The overall promoter methylation is low for *SDHC* gene (*β*-value=0.03) and high for *SDHD* gene (*β*-value=0.89).

Based on available information for 466 tumor samples from TCGA THCA, we then examined whether any demographic or clinical characteristics were associated with *SDHx* gene expression differences. Only earlier age at diagnosis and higher pTNM stage but not others were associated with lower expression of the *SDHx* genes overall (Table 2). Classic PTC histology was overrepresented among patients with low overall *SDHx* expression, compared with those with other histologic types, especially the follicular variant form of PTC. However, no association was seen with residual tumor or final vital status.

## Discussion

Defects in mitochondrial function have long been shown to contribute to the development and progression of cancer. The ‘oncocytic tumors’ theory considers genes encoding proteins with mitochondrial function as putative cancer-associated genes. Supporting this were pilot observations of an increased prevalence of PTC and renal cell carcinoma, both considered oncocytic tumors, in *SDHx* variant carriers in CS/CS-like cases. We are aware that some of the variants we identified in our current study are reported in public databases such as dbSNP and ESP where the frequency varies among different ethnicity background population. This is the reason why we used our ethnicity-matched control as a comparison. The occurrence of these variants in patients with DTC but not in ethnicity-matched controls or sporadic breast cancer is further assurance. *SDHB* A3G (rs11203289) was reported in dbSNP but only in the African–American population, while our samples are derived from white individuals of European ancestry. The most frequent variants *SDHB* S163P (rs33927012), *SDHD* G12S (rs34677591), and *SDHD* H50A (rs11214077) have also been reported in the database. Although these relatively common (1–5% frequency) variants were computationally predicted to be functionally benign (Bayley 2011), our previous experimental data already provided molecular evidence that they could have functional impact in cellular signaling regulation (Ni *et al*. 2008, 2012). The reason why bioinformatics analysis of prediction fails in *SDHx* genes is because they are extremely well conserved throughout species (Ng & Henikoff 2006). Indeed, these very same bioinformatics tools predicted that the *SDHD* P81L missense mutation would be benign when this is really the North American founder mutation predisposing to familial paraganglioma (Baysal *et al*. 2002). With enormous numbers of variations uncovered by whole-genome sequencing, it is essential to realize that functional analysis and clinical correlations must be performed to define the true pathogenic effect of DNA variations (Ni & Eng 2011). In our 2008 study, we showed clear one-to-one correlation between the identified *SDHB* or *SDHD* variants and profound functional phenotypes such as altered reactive oxygen species (ROS) and up regulation of AKT (also known as protein kinase B) and mitogen-activated protein kinase (MAPK). The latter may explain why *SDHB* or *SDHD* variation can result in phenotypes similar to (but not identical to) Cowden and Cowden-like syndromes: the AKT and MAPK pathways are important pathways downstream of PTEN also (Ni *et al*. 2008). In our 2012 study, we validated that germline *SDHx* variants are associated with elevated thyroid cancer risks in Cowden and Cowden-like individuals. We also provided further functional evidence of these germline *SDHx* variants. We showed that these variants led to mitochondrial metabolite imbalance, and in turn cause stabilization of HIF1α, and decreased baseline p53 levels mediated by the noncanonical NQO1 pathway. These functional consequences are at least partially responsible for ROS generation. The cross-talk between SDH and PTEN results in multi-signaling pathways that contribute to tumorigenesis (Ni *et al*. 2012). A recent *in vitro* study specifically in thyroid cancer cell lines also showed that *SDHD* G12S and *SDHD* H50R variants lead to impaired PTEN function through alteration of its subcellular localization accompanied by resistance to apoptosis and induction of migration, mediated by Rous sarcoma protooncogene (SRC) (Yu *et al*. 2014). Taking all the evidence together, we believe that the variants we reported in this and previous studies are very likely associated with thyroid carcinogenesis.

The somatic 1q duplication spans almost 92 Mb, which is not reported in any structural variation or CNV databases. There are more than 100 genes, including *SDHC*, residing in this region. In the TCGA dataset, the 25 samples with somatic *SDHC* duplication had ∼1.5-fold *SDHC* gene expression compared with samples without duplication, indicating that the additional copy of the gene does generate transcript (Supplementary Figure 1, see section on supplementary data given at the end of this article). However, how exactly this large duplication (including *SDHC* and other genes) impacts tumorigenesis warrants further investigation, given that the TCGA thyroid tumor dataset as a whole showed an overall decrease of *SDHC* expression compared with their normal tissues.

The association of germline loss-of-function mutations in *SDHx* genes and loss of SDH subunit protein expression in paraganglioma–pheochromocytoma and gastrointestinal stromal tumors are well established (Neumann *et al*. 2002, 2004, Doyle *et al*. 2012, Dwight *et al*. 2013). At the expression level, most studies utilized immunohistochemistry to measure SDHA and SDHB protein, and showed loss of SDHB in pheochromocytoma–paraganglioma tumors (van Nederveen *et al*. 2009). Reduced SDHB protein expression was also associated with growth and de-differentiation of colorectal cancer cells (Zhang *et al*. 2013). In the study from Papathomas *et al*. (2014), SDHB immunonegativity was observed in renal cell carcinoma but not PTC associated with SDH-related pheochromocytoma/paraganglioma syndrome. In our study with sporadic PTC, we did not see alterations in *SDHB* at the mRNA level, but rather significant reductions in *SDHC/D* gene expression. The methylation analysis in these two genes did not show a difference in tumors compared with normal tissues, indicating the loss of the gene expression was not the result of DNA hypermethylation but most likely due to other transcriptional regulation alterations in tumor cells. As SDHC/D subunits mainly function as the anchor proteins to position the whole SDH complex into the mitochondrial inner membrane, it is likely that the loss of the structural SDHC/D subunits will affect the overall stability and integrity of complex II and lead to mitochondrial abnormalities. It has been reported that the protein assembly of the SDH complex is critical for regulation of cell death, especially the dissociation of SDHA and SDHB subunits from the membrane-anchoring proteins through pH changes or mitochondrial Ca^2^^+^ influx (Hwang *et al*. 2014). Unfortunately, we could not find reliable SDHC/D antibodies to measure protein expression of these two subunits in our tumor samples. The association of overall low *SDHx* gene expression with earlier disease onset as well as higher pTNM stage was found in the TCGA dataset but needs to be verified in an independent study. Even though we did not further investigate the *SDHx* expression in breast tumors, loss of SDHA or SDHB expression by immunohistochemistry has been reported in about 3% of breast cancers and low SDHA/B expression status in breast tumor cells was associated with younger age at diagnosis and low-grade histology (Kim *et al*. 2013).

The question of if and how *SDHx* alterations differ in papillary and follicular histological subtype of thyroid cancer remains to be explored in depth. Our earlier analysis revealed elevated risks of FTC due to germline *PTEN* pathogenic mutations and of PTC for germline *SDHx* alterations in CS/CS-like individuals (Ngeow *et al*. 2011). In this study of sporadic DTCs, germline *SDHx* variations were detected in both PTC and FTC cases. It is notable that *SDHB* transcript levels are decreased in our FTC, albeit represented by small sample size, in contrast to no change in *SDHB* expression in the PTC samples. As the TCGA dataset is limited by its papillary histology-focused sample collection, whether somatic *SDHB* expressional differences could distinguish papillary from follicular histology needs further validation with an expanded sample size. SDHB protein expression has not been examined in sporadic differentiated thyroid tumors by immunohistochemistry yet and it will be interesting to correlate protein expression with its gene expression. Previous microarray analysis reported five genes (*CITED1, CLDN10, IGFBP6, CAV1,* and *CAV2*) that collectively distinguish the two histologic types (Aldred *et al*. 2004). If the differential *SDHB* gene expression was validated, it will be noteworthy to see whether *SDHB* could improve the five-gene classification capability.

In conclusion, we have shown germline and somatic *SDHx* variants occur in sporadic DTC and overall loss of *SDHx* gene expression could represent a molecular signature of differentiated thyroid tumors.

## Supplementary data

This is linked to the online version of the paper at http://dx.doi.org/10.1530/ERC-14-0537.

Supplementary Data

## Figures and Tables

**Table 1:** Germline SDHx variants in 3 independent series of sporadic differentiated thyroid cancer samples and pooled summary

	**Variation**	***n***
(A) Germline *SDHx* variants in *PTEN* mutation-negative DTC individuals (15/241, 6.2%)		
*SDHB* (*n*=6)	c.8C>G, p.Arg3Gly	1
	c.487T>C, p.Ser163Pro	5
*SDHD* (*n*=9)	c.34G>A, p.Gly12Ser	6
	c.149A>G, p.His50Arg	3
(B) Germline *SDHx* variants in TCGA THCA individuals (28/476, 5.9%)		
*SDHB* (*n*=13)	c.8C>G, p.Arg3Gly	1
	c.158G>A, p.Gly53Glu	1
	c.178A>G, p.Thr60Ala	1
	c.425A>T, p.Asp142Val	1
	c.487T>C, p.Ser163Pro	9
*SDHD* (*n*=15)	c.34G>A, p.Gly12Ser	10
	c.149A>G, p.His50Arg	5
(C) Germline *SDHx* variants in CHTN-paired thyroid tumor samples (5/37, 13.5%)		
*SDHB* (*n*=3)	c.8C>G, p.Arg3Gly	2
	c.487T>C, p.Ser163Pro	1
*SDHD* (*n*=2)	c.149A>G, p.His50Arg	2
(D) Germline *SDHx* variants in pooled 754 individuals (48/754, 6.3%)		
*SDHB* (*n*=22)	c.8C>G, p.Arg3Gly	4
	c.158G>A, p.Gly53Glu	1
	c.178A>G, p.Thr60Ala	1
	c.425A>T, p.Asp142Val	1
	c.487T>C, p.Ser163Pro	15
*SDHD* (*n*=26)	c.34G>A, p.Gly12Ser	16
	c.149A>G, p.His50Arg	10

(A) Consecutive series of apparently sporadic *PTEN* mutation-negative differentiated thyroid cancer (DTC) samples from OSU thyroid center; (B) TCGA thyroid cancer (THCA) samples; (C) CHTN paired thyroid tumor-normal tissue samples; (D) pooled 754 subjects from above three sources.

**Table 2:** Demographic and clinical characteristics of TCGA THCA samples based on *SDHx* gene expression differences

**Gene_score**^a^	**0**	**1**	**2**	**3**	**4**	***P* value**^b^
Mean of age_at_diagnosis (*n*)	44.98 (*n*=245)	47.37 (*n*=104)	50.14 (*n*=63)	50.60 (*n*=25)	52.93 (*n*=29)	0.015
Gender						
Female (*n*=343)	178	77	45	20	23	0.867
Male (*n*=123)	67	27	18	5	6	
Pathology_T						
T1 (*n*=135)	75 (55.6%)	22 (16.3%)	22 (16.3%)	7 (5.2%)	9 (6.7%)	0.051
T2 (*n*=154)	83 (53.9%)	38 (24.7%)	15 (9.7%)	3 (1.9%)	15 (9.7%)	
T3 (*n*=156)	76 (48.7%)	40 (25.6%)	22 (14.1%)	14 (9%)	4 (2.6%)	
T4 (*n*=19)	9 (47.4%)	4 (21.1%)	4 (21.1%)	1 (5.3%)	1 (5.3%)	
Pathology_N						
N0 (*n*=213)	106 (49.8%)	38 (17.8%)	38 (17.8%)	16 (7.5%)	15 (7.0%)	<0.001
N1 (*n*=207)	119 (57.5%)	57 (27.5%)	19 (9.2%)	7 (3.4%)	5 (2.4%)	
NX (*n*=46)	20 (43.5%)	9 (19.6%)	6 (13.0%)	2 (4.3%)	9 (19.6%)	
Pathology_M						
M0 (*n*=252)	147 (58.3%)	51 (20.2%)	39 (15.5%)	10 (4.0%)	5 (2.0%)	<0.001
M1 (*n*=8)	8 (100%)	0 (0%)	0 (0%)	0 (0%)	0 (0%)	
MX (*n*=205)	90 (43.9%)	53 (25.9%)	24 (11.7%)	14 (6.8%)	24 (11.7%)	
NA (*n*=1)	0 (0%)	0 (0%)	0 (0%)	1 (100%)	0 (0%)	
Pathology_stage						
Stage I (*n*=264)	145 (54.9%)	59 (22.3%)	37 (14.0%)	10 (3.8%)	13 (4.9%)	0.002
Stage II (*n*=0)	0	0	0	0	0	
Stage III (*n*=104)	50 (48.1%)	29 (27.9%)	13 (11.5%)	11 (10.6%)	2 (1.9%)	
Stage IV (*n*=7)	6 (85.7%)	1 (14.3%)	0 (0%)	0 (0%)	0 (0%)	
NA (*n*=91)	44 (48.4%)	15 (16.5%)	14 (15.4%)	4 (4.4%)	14 (15.4%)	
Histology						
Classic PTC (*n*=321)	189 (58.9%)	73 (22.7%)	38 (11.8%)	11 (3.4%)	10 (3.1%)	<0.001
FvPTC (*n*=99)	36 (36.4%)	17 (17.2%)	16 (16.2%)	12 (12.1%)	18 (18.2%)	
TallCell PTC (*n*=35)	14 (40.0%)	12 (34.3%)	8 (22.9%)	1 (2.9%)	0 (0%)	
Others (*n*=11)	6 (54.5%)	2 (18.2%)	1 (9.1%)	1 (9.1%)	1 (9.1%)	

a Gene_score (range from 0 to 4) was calculated by first categorizing each gene's expression as either 1 as higher than normal, or 0 as lower than normal, then adding up the binary score for each of these *SDHA–D* genes.

b *P* value was calculated by one-way ANOVA for age_at_diagnosis with degree of freedom of 4, and by Pearson *χ*^2^ test for the rest of comparisons.
