# Supplementary material for: Germline and somatic SDHx alterations in apparently sporadic differentiated thyroid cancer
Source: Endocr Relat Cancer. 2015 Jan 5;22(2):121–30. doi: 10.1530/ERC-14-0537 (PMC4335266; doi:10.1530/ERC-14-0537)
Supplement: Supplementary Data [file supp_22_2_121__index.html]

Supplementary Data 

# Germline and somatic *SDHx* alterations in apparently sporadic differentiated thyroid cancer

## Supplementary Data

**Files in this Data Supplement:**

- Supplementary Figure 1 - *SDHC* gene expression in samples with somatic SDHC duplication compared to samples without, in TCGA dataset.(PDF 189 KB)
- Supplementary Table 1 - Primers used for SDHB/C/D mutation scanning. (PDF 9 KB)
- Supplementary Table 2 - Somatic chromosome1 duplications in TCGA THCA dataset. (PDF 9 KB)
- Supplementary Table 3 - *PTEN* and *SDHx* gene expression correlation in TCGA THCA dataset. (PDF 12 KB)
