# Supplementary figures and images for: Germline and somatic SDHx alterations in apparently sporadic differentiated thyroid cancer
Source: Endocr Relat Cancer. 2015 Jan 5;22(2):121–30. doi: 10.1530/ERC-14-0537 (PMC4335266; doi:10.1530/ERC-14-0537)

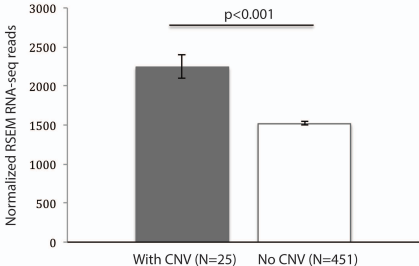

Supplement: Supplementary Data [file supp_22.2.121_Supplementary_figure_1.pdf]
