# Supplementary material for: Germline and somatic SDHx alterations in apparently sporadic differentiated thyroid cancer
Source: Endocr Relat Cancer. 2015 Jan 5;22(2):121–30. doi: 10.1530/ERC-14-0537 (PMC4335266; doi:10.1530/ERC-14-0537)
Supplement: Supplementary Data [file supp_22.2.121_Supplementary_table_1.pdf]

Supplemental Table 1. *SDHx* mutation screening LightScanner primer list

Name	Sequence (5' to 3')
SDHB_1F	GCGACCTCGGGGTTAAG
SDHB_1R	TCCCTCTCTGAGGCTCCA
SDHB_2F	CCAGCAAAATGGAATTATCTTGTATTTCTA
SDHB_2R	CTCCTTCAATAGCTGGCTT
SDHB_3F	GGTTGAACGTTACATAAATACCACT
SDHB_3R	TATCTGGAGCCCAACAGGA
SDHB_4F	GGATATGGGTGAGGATGTGTTAAA
SDHB_4R	TAGCGTAACACACATAGCACTG
SDHB_5F	GTGATGATGGAATCTGATCCTTT
SDHB_5R	AATTCTTCAGATTGAAACAATAAATAGGG
SDHB_6F	AGCTCCTGCCTCTCTTT
SDHB_6R	GGATGCTTGAGTTTCAATTTCTCTTA
SDHB_7F	CAGCTAATCATCCCTGGTT
SDHB_7R	CTCTGAGGCAGAGCTGAG
SDHB_8F	CAGTTTCAGTTATCATTTGTTATTGATTC
SDHB_8R	CTGAGCTGGTTATAAATCATGTTTAG
SDHC_1F	AGAGACTCTCGTCACATGACA
SDHC_1R	ACTCCCAGTCCCACTGAA
SDHC_2F	ATACTAAAGTTGATCTCTAAATGTGTATTG
SDHC_2R	AATAAATAATCTCCAGACTTAGAAACTTAC
SDHC_3F	TTAGTTATTTTCAAACGGTCTGGT
SDHC_3R	TCTCTGGCTCCAGAATCC
SDHC_4F	GATAGACTCTCTACTATGGTGTCA
SDHC_4R	AAAGAAGCACATATGTGTGTGTAA
SDHC_5F	CTGTGACAAGCTACTTGGTT
SDHC_5R	CTTCACAGAGAAAATGTGCAAATC
SDHC_6F	GAACTGTTAATGTCCTATTTACTGAAATTC
SDHC_6R	CAGGTACTCTACTGCTCCAAG
SDHD_1F	TAAGTGGTTCCGGGTTGG
SDHD_1R	CACCTCAGGGTGGGAAG
SDHD_2F	CCTGTTAAAGGAGAGGTTCTTAT
SDHD_2R	CAGAAAGCAGCAGCGAT
SDHD_3F	TGTGTGTTTCTCACATCAACTTT
SDHD_3R	ACTGAGCAGACAACTATATTTGG
SDHD_4F	GTATAGTCTTCTAATTTCACTGTGGT
SDHD_4R	GCAAAGAGGCATACATCAATTC
