# Supplementary material for: Germline and somatic SDHx alterations in apparently sporadic differentiated thyroid cancer
Source: Endocr Relat Cancer. 2015 Jan 5;22(2):121–30. doi: 10.1530/ERC-14-0537 (PMC4335266; doi:10.1530/ERC-14-0537)
Supplement: Supplementary Data [file supp_22.2.121_Supplementary_table_2.pdf]

Supplemental Table 2. List of somatic duplication regions in chromosome 1 from TCGA THCA samples

Chr	Start	End	#Probe	Sample
1	3218610	247813706	129135	A3I5
1	120523902	247813706	61339	A3MZ
1	120527361	247813706	61319	A40L
1	149879545	163164485	7381	A3GU
1	149879545	196004553	28325	A192
1	149879545	237413292	54817	A13U
1	149879545	239264404	56403	A3O3
1	149879545	247813706	61287	A3ZO
1	149881398	165391382	9039	A4MD
1	149881398	197319745	28589	A4EU
1	149881398	247813706	61316	A3T9
1	149881398	247813706	61338	A3O1
1	149881398	247813706	60623	A4IB
1	149881398	247813706	61343	A231
1	149881398	247813706	61253	A3GP
1	149881398	247813706	61249	A3EB
1	149881398	247813706	61296	A290
1	149884927	165391382	8947	A4MD
1	149884927	238649757	55254	A4MC
1	149884927	247813706	60609	A4MB
1	149898951	169949913	12508	A39P
1	150033345	247813706	61008	A4B0
1	154609657	247813706	59157	A3NM
1	158514944	220761607	39966	A3H5
1	158728389	215338184	35794	A4JX
1	3218610	247813706	129135	A3I5
1	120523902	247813706	61339	A3MZ
1	120527361	247813706	61319	A40L
1	149879545	163164485	7381	A3GU
1	149879545	196004553	28325	A192
1	149879545	237413292	54817	A13U
1	149879545	239264404	56403	A3O3
1	149879545	247813706	61287	A3ZO

1	149881398	165391382	9039	A4MD
1	149881398	197319745	28589	A4EU
1	149881398	247813706	61316	A3T9
1	149881398	247813706	61338	A3O1
1	149881398	247813706	60623	A4IB
1	149881398	247813706	61343	A231
1	149881398	247813706	61253	A3GP
1	149881398	247813706	61249	A3EB
1	149881398	247813706	61296	A290
1	149884927	165391382	8947	A4MD
1	149884927	238649757	55254	A4MC
1	149884927	247813706	60609	A4MB
1	149898951	169949913	12508	A39P
1	150033345	247813706	61008	A4B0
1	154609657	247813706	59157	A3NM
1	158514944	220761607	39966	A3H5
1	158728389	215338184	35794	A4JX
