# Supplementary material for: Germline and somatic SDHx alterations in apparently sporadic differentiated thyroid cancer
Source: Endocr Relat Cancer. 2015 Jan 5;22(2):121–30. doi: 10.1530/ERC-14-0537 (PMC4335266; doi:10.1530/ERC-14-0537)
Supplement: Supplementary Data [file supp_22.2.121_Supplementary_table_3.pdf]

Supplemental Table 3. Statistical correlation between *PTEN*, *SDHA, SDHB, SDHC*, and *SDHD* gene expression from TCGA THCA data

Correlations

		PTEN	SDHA	SDHB	SDHC	SDHD
PTEN	Pearson Correlation	1	.157**	.072	.247**	.187**
	Sig. (2-tailed)		.001	.121	.000	.000
	N	466	466	466	466	466
SDHA	Pearson Correlation	.157**	1	.864**	.609**	.693**
	Sig. (2-tailed)	.001		.000	.000	.000
	N	466	466	466	466	466
SDHB	Pearson Correlation	.072	.864**	1	.617**	.730**
	Sig. (2-tailed)	.121	.000		.000	.000
	N	466	466	466	466	466
SDHC	Pearson Correlation	.247**	.609**	.617**	1	.681**
	Sig. (2-tailed)	.000	.000	.000		.000
	N	466	466	466	466	466
SDHD	Pearson Correlation	.187**	.693**	.730**	.681**	1
	Sig. (2-tailed)	.000	.000	.000	.000	
	N	466	466	466	466	466

**. Correlation is significant at the 0.01 level (2-tailed).
